# Mathematical Model for Small Size Time Series Data of Bacterial Secondary Metabolic Pathways

**DOI:** 10.1177/1177932218775076

**Published:** 2018-05-16

**Authors:** Daisuke Tominaga, Hideo Kawaguchi, Yoshimi Hori, Tomohisa Hasunuma, Chiaki Ogino, Sachiyo Aburatani

**Affiliations:** 1 Computational Bio Big-Data Open Innovation Laboratory (CBBD-OIL), National Institute of Advanced Industrial Science and Technology (AIST), Waseda University, Tokyo, Japan; 2 Graduate School of Science, Technology and Innovation, Kobe University, Kobe, Japan; 3 Organization of Advanced Science and Technology, Kobe University, Kobe, Japan; 4 Graduate School of Engineering, Kobe University, Kobe, Japan

**Keywords:** Metabolic pathway, pathway dynamics, small sample, S-system, Michaelis-Menten

## Abstract

Measuring the concentrations of metabolites and estimating the reaction rates of each reaction step consisting of metabolic pathways are significant for an improvement in microorganisms used in maximizing the production of materials. Although the reaction pathway must be identified for such an improvement, doing so is not easy. Numerous reaction steps have been reported; however, the actual reaction steps activated vary or change according to the conditions. Furthermore, to build mathematical models for a dynamical analysis, the reaction mechanisms and parameter values must be known; however, to date, sufficient information has yet to be published for many cases. In addition, experimental observations are expensive. A new mathematical approach that is applicable to small sample data, and that requires no detailed reaction information, is strongly needed. S-system is one such model that can use smaller samples than other ordinary differential equation models. We propose a simplified S-system to apply minimal quantities of samples for a dynamic analysis of the metabolic pathways. We applied the model to the phenyl lactate production pathway of *Escherichia coli*. The model obtained suggests that actually activated reaction steps and feedback are inhibitions within the pathway.

## Introduction

### Mathematical model for metabolic pathways

The artificial and industrial uses of microorganisms for material production have a long history of more than a thousand years. Recently, genetic operations have been widely applied to improve production. Two generally considered approaches introduce enzymes that have higher activities from other organisms or species and introduce enzymes to realize metabolic pathways that do not naturally occur in the microorganisms. The former method is popular because its operation is simpler and improvements are more predictable than with the latter method. Conventional gene modifications using ultraviolet or other radiation types are easy to achieve and have been widely applied in many industries. Nevertheless, the efficiency of such improvements is quite low because gene modifications occur accidentally and uncontrollably, and progress is made serendipitously. Therefore, gene introduction is currently used along with conventional methods.

Target genes for modification are chosen based on information including the reaction rates of the respective reaction steps within the metabolic pathway and include the production materials and substrates of microorganisms, as well as changes in the reaction rates through changes in the concentrations of the metabolites that consist of the pathway. Bottleneck reaction steps and feedback loop inhibitions are suggested based on this information. The genes of enzymes used in such reactions are candidates for modification.

The rates of enzymic reactions are generally defined as the limit of changes in the compounds over time.^1,2^ Several formulae are established for the types of enzymic reactions, such as inhibition schemes. The most popular is the Michaelis-Menten law^3,4^ for a simple one-to-one enzymic reaction without any inhibition or catalysis using an enzyme. The reaction rate is modeled based on the following reaction scheme:

(1) S+E⇌ES→P+E

in which S indicates the substrate of the reaction, E signifies the enzyme, and P denotes the product. A bidirectional arrow represents a reversible reaction. A 1-directional arrow signifies a 1-way reaction. The reaction rate is modeled as the following ordinary differential equation (ODE):

(2) $$\frac{d[P]}{dt}=\frac{V_{\max}[S]}{K_{m}+[S]}$$

Therein, a pair of square brackets denotes the concentration of the compound, t is the time, and $V_{\max}$ and $K_{m}$ are parameters that define the kinetic characteristic of the enzyme. All reaction steps in a metabolic pathway can be represented using the ODE above if all reactions are simple enzymic reactions and if the parameter values are defined. The pathway is then modeled as simultaneous ODEs or the ODE system. Consequently, simulations can be done of concentration changes of metabolites, stability analysis, steady-state estimation, and bottleneck finding.^5,6^

Generally, finding the parameter values is difficult and expensive because it requires enzyme isolation and measurements of the reaction rates in test tubes (in vitro measurements). Although the amount of enzyme information in the literature and public databases is growing,^7^ the values of the kinetic parameters have not been sufficiently published or accumulated. An enzyme generally has different parameter values for the various conditions and species of organisms. Moreover, the parameter values generally differ between in vitro and in vivo (in living cells) conditions.^8,9^ For most industrial applications, a dynamical analysis of the pathways must be conducted without reaction scheme information or kinetic parameter values.

### Canonical ODE model

The ODE systems in canonical forms are applicable because they are independent of the molecular mechanism of the reaction scheme. The S-system^6^ is one such canonical ODE model. For a reaction scheme with 2 reactions, the following is used:

(3) $$X_{1}+X_{2}+\cdots +X_{l}\to P\to X_{l+1}+X_{l+2}+\cdots +X_{m}$$

where $X_{1}$, $X_{2}\ldots$, and $X_{l}$ and P denote the substrates and product of the first reaction, respectively, and P and $X_{l+1},\ldots ,X_{m}$ denote the substrate and products of the second reaction, respectively. The S-system form is represented as follows:

(4) $$\frac{d[P]}{dt}=\alpha \prod_{j=1}^{n}{\left[X_{j}\right]}^{g_{j}}-\beta \prod_{j=1}^{n}{\left[X_{j}\right]}^{h_{j}}$$

where $X_{j}$ indicates the concentration of the metabolite j, $g_{j}$ signifies the kinetic parameter representing the influence of $X_{j}$ to the increasing processes of P, $h_{j}$ denotes the influence of $X_{j}$ on the decreasing processes of P, and α and β are rate constants. The first term of the left-hand side of the equation represents the total rate of the increasing or composing processes of P. The second term is the total rate of the decreasing or decomposing processes. In addition, l and m in the reaction (3) are the numbers of composing and decomposing processes of P, respectively. All l and m compounds are suffixed sequentially in Equation (4).

The S-system above is a simplified form of the general mass action law,^6^ which can be presented as follows:

(5) $$\frac{d[P]}{dt}=\sum_{i=1}^{m}\alpha_{i}\prod_{j=1}^{n}{\left[X_{ij}\right]}^{g_{ij}}$$

Therein, the i suffix denotes each composing and decomposing reaction of P. A simplification of the S-system summarizes the composing reactions of P into a term using α and the decomposing reactions into a β term in Equation (4). The parameters $g_{j}$ and $h_{j}$ correspond to reaction orders in the mass action law, $g_{ij}$, respectively, and indicate dependencies between the metabolites P and $X_{j}$. Consequently, they represent the network scheme of the reaction pathway. No direct dependence exists between P and $X_{j}$ when $g_{j}$ and $h_{j}$ in Equation (4) are equal to 0. $X_{j}$ suppresses the production of P when $g_{j}$ is negative.

The parameter values of $g_{j}$, $h_{j}$, α, and β can be estimated using numerical optimization methods for finding the parameter values by which the calculated time series of P through a numerical integration of Equation (4) matches the observed time series of the concentration of P. The determined values of $g_{j}$ and $h_{j}$ might be considered as representing orders of each reaction between $X_{j}$ and P. However, parameter optimization is an inverse problem^10^ because several sets of different parameter values are generally found for the given observed time series data. Restrictions and limitations are effective for difficulties such as fixing some $g_{j}$ to 0 based on biological knowledge.

### Method for small sample

Numerical optimizations require a sufficient number of observed samples. Smaller needs are better because observations entail a certain amount of costs. A mathematical model with fewer parameters requires fewer samples. We propose a canonical ODE model for small samples through the simplification of the second term of Equation (4), as shown below:

(6) $$\frac{d[P]}{dt}=\alpha \prod_{j=1}^{n}{\left[X_{j}\right]}^{g_{j}}-\beta {\left[P\right]}^{h}$$

By the mass action law, the decomposition rate of a compound depends solely on its concentration in many biological processes, such as Michaelis-Menten–type reactions shown in Equation (2). We introduce this idea as an assumption in Equation (6). Although decomposition reactions are often modeled as linear ODE, such as

$$\frac{d[P]}{dt}=A\exp(-[P]t)$$

our model includes a nonlinear decomposition term because we suspect that a linear term might be too simple for the metabolite in a complex biological network system containing many unknown reactions. Our assumption is reasonable when regulation of the degradation processes by these unknown reactions is not significant.

The resulting time series of the model based on a numerical integration varies greatly through a change in the initial value. Finding the best initial value is difficult because the observed initial value frequently has errors, particularly for small sample data sets. Therefore, we compare the model and data in differential spaces. The differential of the observed data can be calculated through a numerical differentiation, and the parameter values can be evaluated by comparing the differential values with the values of Equation (6).

We evaluated the proposed method according to its application to the phenyl lactate (PL) production pathway from glucose using *Escherichia coli*. For a pathway that includes branches and feedback loops, we estimated the actually activated reaction steps and activities of the feedback inhibitions suggesting strategies for an improvement in production.

## Method

First, we build a pathway map based on information from the literature and different databases. We then choose some metabolites in the map as observation targets. A pathway map is reconstructed using only the target metabolites. The observations are measurements of the target metabolite concentration at sampling time points with equal intervals. A simplified S-system model is defined based on the reconstructed pathway map by fixing some $g_{j}$ to 0 if the link to P from $X_{j}$ does not exist in the reconstructed pathway map.

**Figure 1.**
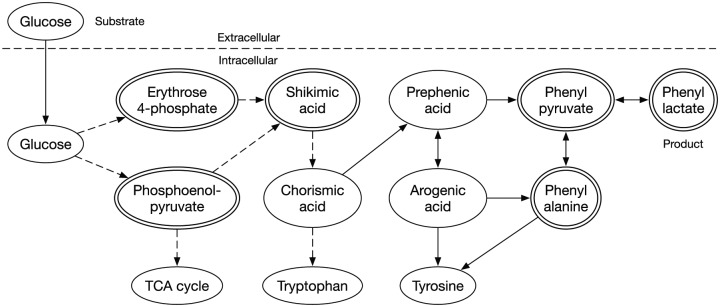
Main pathway of phenyl lactate metabolism of *Escherichia coli*. A dashed line indicates that the path consists of plural reactions. A solid 1-directional line indicates a single enzymic reaction. A bidirectional line indicates 2 reactions: forward and backward processes catalyzed by the same or different enzymes. A double-lined circle indicates a target metabolite selected for observation.

**Figure 2.**
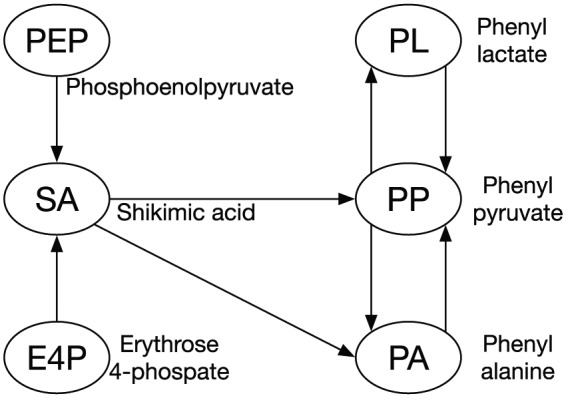
Reconstructed pathway consisting of observation target metabolites.

**Figure 3.**
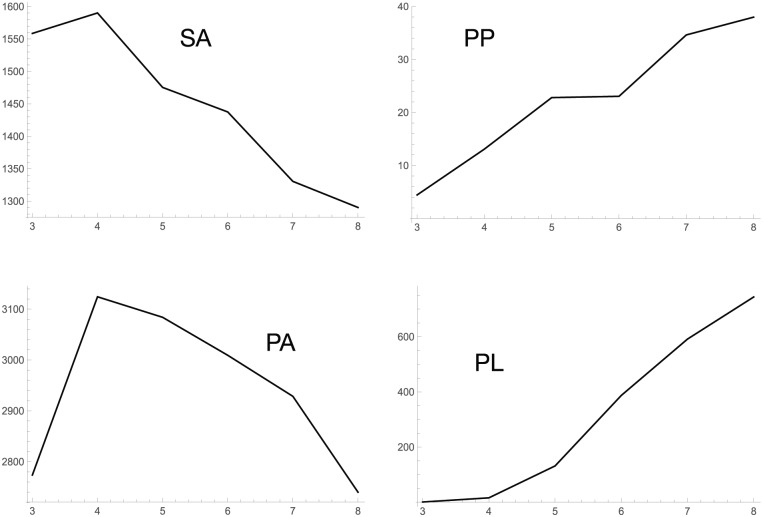
Observed time series of concentrations of target metabolites at the 6 time points. SA, PP, PA, and PL indicate shikimic acid, phenyl pyruvate, phenylalanine, and PL, respectively.

Observed time series data on the concentration of metabolites are numerically differentiated. Optimal values of $g_{j}$, h, α, and β are sought using a nonlinear numerical optimization method such as a genetic algorithm^10^ or differential evolution^11^ to minimize the difference between differentials of the observed data through a numerical differentiation and those from Equation (6). The optimal $g_{j}$ is considered, which represents the activity of the reaction from $X_{j}$ to P. A creation of the formula in Equation (6) and an optimization of the parameters are conducted for each target metabolite. We choose the PL production metabolic pathway^12–15^ (Figure 1) as the application target and 6 metabolites for the observation target. Then, we reconstruct the pathway using only the observation target metabolites (Figure 2). Six sampling time points are used. Changes in the concentration of the metabolites are observed in the log phase of the cell growth (Figure 3).

### Pathway map construction and observation

The pathway from glucose to PL consists roughly of glycolysis and shikimic acid (SA) pathways. Phenyl lactate is produced from phenyl pyruvate (PP), PP is from phenylalanine (PA), and PA is from prephenic acid. In addition, prephenic acid is from chorismic acid in the SA pathway. Choosing phosphoenolpyruvate (PEP), erythrose 4-phosphate (E4P), SA, PA, PP, and PL as the observation targets, we then reconstruct the pathway for these 6 metabolites (Figure 2). The pathway includes a branching point and 2 feedback loops.

### ODE models for the respective metabolite

Changes in concentration of metabolites in the reconstructed pathway are modeled mathematically using the following ODE models:

(7) $$\begin{array}{l} \frac{d[SA]}{dt}=\alpha_{SA}{\left[PEP\right]}^{g_{SA,PEP}}{\left[E4P\right]}^{g_{SA,E4P}}\\ \;\;\;\;\;\;\;\;\;\;\;\;\;\;\;\;\;\;\;-\beta_{SA}{\left[SA\right]}^{h_{SA}}\\ \frac{d[PP]}{dt}=\alpha_{PP}{\left[SA\right]}^{g_{PP,SA}}{\left[PA\right]}^{g_{PP,PA}}{\left[PL\right]}^{g_{PP,PL}}\\ \;\;\;\;\;\;\;\;\;\;\;\;\;\;\;\;\;\;\;-\beta_{PP}{\left[PP\right]}^{h_{PP}}\\ \frac{d[PA]}{dt}=\alpha_{PA}{\left[SA\right]}^{g_{PL,SA}}{\left[PP\right]}^{g_{PA,PP}}-\beta_{PA}{\left[PA\right]}^{h_{PA}}\\ \frac{d[PL]}{dt}=\alpha_{PL}{\left[PP\right]}^{g_{PL,PP}}-\beta_{PL}{\left[PL\right]}^{h_{PL}} \end{array}$$

Actually, production of PEP and E4P is not controlled by any other metabolites, and these compounds are independent variables in the ODE system above. Their respective dynamics are not modeled. Parameter $g_{PP,SA}$ in the ODE system represents the summarized actual activity of the reaction chain to PP that is produced from SA, consisting of several reaction steps. Here, $g_{a,b}$ indicates that the rate of change in the concentration of a is increased by b when the sign of $g_{a,b}$ is positive. A negative value of $g_{a,b}$ means that b suppresses the composition processes of a. A larger h signifies a higher rate of decomposition or consumption of the metabolite. A negative h means that the metabolite suppresses the decomposition itself. Therein, α and β are fixed rate coefficients. For each metabolite, the activities of the respective reaction steps of the composition of the metabolite can be compared.

Time differential values of the metabolite concentration are calculable using the ODE system by determining all parameter values. We introduce the differential evolution algorithm^11^ to find the parameter values that minimize the differences between the differential of the concentration values calculated using the ODE system, as shown below:

(8) $$E=\sum_{t=1}^{T}\left(D_{ct}-D_{ot}\right)$$

Therein, E indicates the summarized differences, $D_{ct}$ signifies the differentials calculated by the ODE system with the parameter values at time point t, and $D_{ot}$ denotes the differential at time point *t* of the observed data. Here, $D_{ot}$ is calculated using the quadratic interpolation of the observed data, and E is minimized by searching the parameter values of g, h, α, and β for each metabolite. The optimization algorithm introduces uniformly distributed random numbers for the initial values of the search parameters.

## Result

### Model interpretation

**Figure 4.**
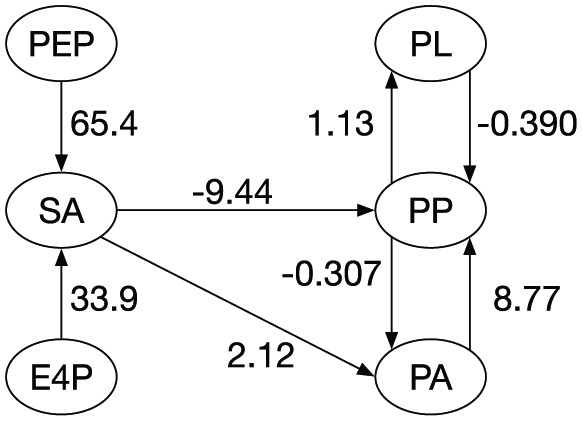
Estimated actual reaction activities on the reconstructed pathway. Numerical values shown in the figure are $g_{j}$ in Equation (6), which represent incoming links for each metabolite. SA, PP, PA, and PL indicate shikimic acid, phenyl pyruvate, phenylalanine, and PL, respectively.

The ODE system with the determined parameter values is shown below (Figure 4):

$$\begin{array}{c} \frac{d[SA]}{dt}=21.1{\left[PEP\right]}^{65.4}{\left[E4P\right]}^{33.9}-99.2{\left[SA\right]}^{-0.0674}\\ \frac{d[PP]}{dt}=9.38{\left[SA\right]}^{-9.44}{\left[PA\right]}^{8.77}{\left[PL\right]}^{-0.390}\\ -57.2{\left[PP\right]}^{-1.95}\\ \frac{d[PA]}{dt}=0.000223{\left[SA\right]}^{2.12}{\left[PP\right]}^{-0.307}\\ -90.7{\left[PA\right]}^{0.207}\\ \frac{d[PL]}{dt}=5.60{\left[PP\right]}^{1.13}-1.51\times 10^{-6}{\left[PL\right]}^{2.82} \end{array}$$

The ODE system suggests that the reaction chain to SA from PEP is more active than that from E4P. In addition, PP production is inhibited by feedback loops from both PA and PL.

There are 3 negative value parameters. Reaction steps that are represented by these parameters consist of only productive enzymic reactions in the literature.^12,13^ These negative parameters may suggest unknown regulatory pathways whose effects look substrate inhibitions.

## Discussion

### Numerical advantages of our model

The changes in metabolite concentration over time can be modeled using the Michaelis-Menten law, as shown below:

$$\begin{array}{c} \frac{d[SA]}{dt}=\frac{V_{SA,PEP}[PEP]}{K_{SA,PEP}+[PEP]}+\frac{V_{SA,E4P}[E4P]}{K_{SA,E4P}+[E4P]}\\ -\frac{V_{PP,SA}[SA]}{K_{PP,SA}+[SA]}-\frac{V_{PA,SA}[SA]}{K_{PA,SA}+[SA]}\\ \frac{d[PP]}{dt}=\frac{V_{PP,SA}[SA]}{K_{PP,SA}+[SA]}+\frac{V_{PP,PA}[PA]}{K_{PP,PA}+[PA]}\\ +\frac{V_{PP,PL}[PL]}{K_{PP,PL}+[PL]}\\ -\frac{V_{PA,PP}[PP]}{K_{PA,PP}+[PP]}-\frac{V_{PL,PP}[PP]}{K_{PL,PP}+[PP]}\\ \frac{d[PA]}{dt}=\frac{V_{PA,SA}[SA]}{K_{PA,SA}+[SA]}+\frac{V_{PA,PP}[PP]}{K_{PA,PP}+[PP]}\\ -\frac{V_{PP,PA}[PA]}{K_{PP,PA}+[PA]}\\ \frac{d[PL]}{dt}=\frac{V_{PL,PP}[PP]}{K_{PL,PP}+[PP]}-\frac{V_{PP,PL}[PL]}{K_{PP,PL}+[PL]} \end{array}$$

For each metabolite, there are 8 parameters for SA, 10 for PP, 6 for PA, and 4 for PL because a model of the reaction step (single enzymic reaction) has 2 parameters. Changes of concentrations in time of SA and PP cannot be modeled using the 6 sampling data in this study. There are a total of 16 parameters for the reconstructed pathway because some parameters are common (1 of the outgoing reactions of SA is an incoming reaction of PP). Some numerical optimization methods can search for the 16 parameters simultaneously. However, such a simultaneous nonlinear numerical optimization is not easy, and the difficulty increases significantly with an increase in the number of parameters (“curse of dimensionality”).

The form of the S-system model is reduction or simplification of general mass action (GMA) law model shown as follows:

$$\begin{array}{c} \frac{d[SA]}{dt}=\alpha_{SA}{\left[PEP\right]}^{g_{SA,PEP}}{\left[E4P\right]}^{g_{SA,E4P}}\\ -\beta_{1SA}{\left[SA\right]}^{g_{PP,SA}--\beta_{2SA}g_{PA,SA}}\\ \frac{d[PP]}{dt}=\alpha_{PP}{\left[SA\right]}^{g_{PP,SA}}{\left[PA\right]}^{g_{PP,PA}}{\left[PL\right]}^{g_{PP,PL}}\\ -\beta_{1PP}{\left[PP\right]}^{g_{PA,PP}-\beta_{2PP}g_{PL,PP}}\\ \frac{d[PA]}{dt}=\alpha_{PA}{\left[SA\right]}^{g_{PL,SA}}{\left[PP\right]}^{g_{PA,PP}}-\beta_{PA}{\left[PA\right]}^{g_{PP,PA}}\\ \frac{d[PL]}{dt}=\alpha_{PL}{\left[PP\right]}^{g_{PL,PP}}-\beta_{PL}{\left[PL\right]}^{g_{PP,PL}} \end{array}$$

For each metabolite, there are 7 S-system parameters for SA, 8 for PP, 5 for PA, and 4 for PL, or 18 in total because some of the parameters are the same. The number of parametes are reduced for SA, PP, and PA compared with Michaelis-Menten models; however, concentrations of SA and PP cannot be modeled even when using the GMA. In our proposed model, the corresponding numbers are 5, 6, 5, and 4, or 20 in all, which is not a small number; however, it is not a problem that the total number of parameters is larger than in the S-system because no parameters are common to any 2 metabolites, and the parameters of each metabolite are optimized independently of the other metabolites. Our model has fewer parameters for each metabolite, which indicates that our model is more robust against errors than the S-system and Michaelis-Menten models.

The less number of model parameters means that the model needs a less number of data. Michaelis-Menten model consists of 2 parameters for each reaction, thus 2n parameter values must be determined for a system that consists of n compounds, whereas the simplified S-system contains n+2. This means that the simplified S-system model needs about a half quantity of data compared with a Michaelis-Menten model.

**Figure 5.**
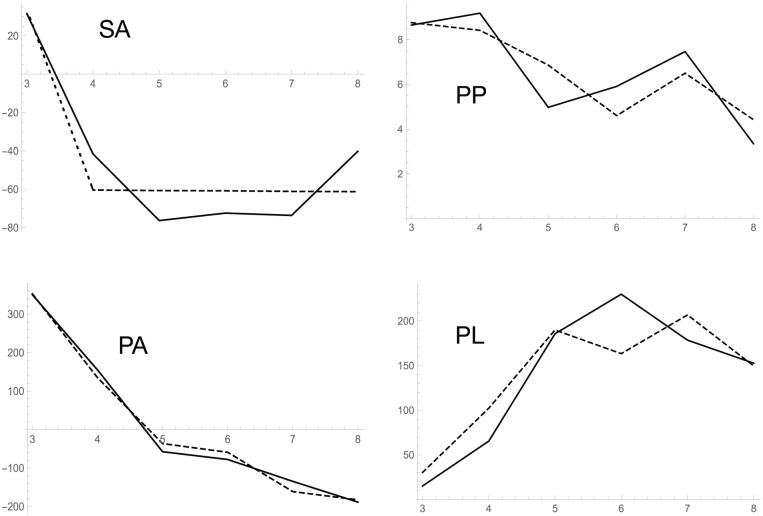
Values of differentials calculated from the observed data through a quadratic interpolation and calculated from the optimized ODE model (simplified S-system model). The dashed line shows differential values calculated from the ODE model with the optimized parameters. A solid line represents the numerical differentiation from the observed data. SA, PP, PA, and PL indicate shikimic acid, phenyl pyruvate, phenylalanine, and phenyl lactate, respectively. Michaelis-Menten model and the full S-system model cannot be obtained due to the much number of parameters of the models compared with the number of data. SA, PP, PA, and PL indicate shikimic acid, phenyl pyruvate, phenylalanine, and PL, respectively.

Although the model and data do not match perfectly because the data generally include errors in the probabilistic distributions, the optimization precision of the parameters (model fitness to the data) shown in Figure 5 is apparently sufficient. In terms of biochemical engineering, the values of some parameters shown in Figure 4 are large as the reaction order. Perhaps because of fluxes of pathways other than the reconstructed pathway from which we omitted pathways other than the main reaction chain, glycolysis has many branches to other subsystems. However, the parameter values for PA (PA in Figure 4), PP, and PL might be more reliable because the reaction steps occurring naturally around these metabolites may be nearly the same as those of the reconstructed pathway.

The parameter values of the α term of a metabolite (of the incoming link of a metabolite in the pathway map) are directly comparable. Phenyl pyruvate has 3 parameters to compare, 2 of which are negative, and 1 of which is positive. Therefore, PP production, which is inhibited by SA and PL (the final product), depends mainly on PA.

Two negative feedback loops exist: PP production is inhibited by PL, and PA production is inhibited by PP. Although it can be readily imagined that the inhibition of the feedback reactions increases the production of PL, the main inhibitory effect on PP production is from the SA. Disrupting one or more genes of the reactions to PP from SA might improve the PL production.

Phenylalanine decreases gradually (Figure 3), but shows no natural decomposition, which is represented as Aexp(−Bt), and might be caused by the incoming link from PP.

In conclusion, the results show that the reliability of the estimated parameter values might not be the best or even very high because the reconstructed pathway and the ODE system are simplified. These values suggest that the target genes can be modified for an industrial improvement in production using microorganisms. This case study presents several suggestions that may be useful when constrained to only a few samples or low observation costs.
